# Effect of Obesity on Arithmetic Processing in Preteens With High and Low Math Skills: An Event-Related Potentials Study

**DOI:** 10.3389/fnhum.2022.760234

**Published:** 2022-03-10

**Authors:** Graciela C. Alatorre-Cruz, Heather Downs, Darcy Hagood, Seth T. Sorensen, D. Keith Williams, Linda J. Larson-Prior

**Affiliations:** ^1^Department of Pediatrics, University of Arkansas for Medical Sciences, Little Rock, AR, United States; ^2^Arkansas Children’s Nutrition Center, Little Rock, AR, United States; ^3^Vice Chair for Education, Department of Biostatistics, Arkansas Children’s Nutrition Center, Little Rock, AR, United States; ^4^Departments of Psychiatry, Neurology, Neurobiology and Developmental Sciences, and Biomedical Informatics, University of Arkansas for Medical Sciences, Little Rock, AR, United States

**Keywords:** obesity, preteens, math skills, P300, arithmetic N400

## Abstract

Preadolescence is an important period for the consolidation of certain arithmetic facts, and the development of problem-solving strategies. Obese subjects seem to have poorer academic performance in math than their normal-weight peers, suggesting a negative effect of obesity on math skills in critical developmental periods. To test this hypothesis, event-related potentials (ERPs) were collected during a delayed-verification math task using simple addition and subtraction problems in obese [above 95th body mass index (BMI) percentile] and non-obese (between 5th and 90th BMI percentile) preteens with different levels of math skill; thirty-one with low math skills (14 obese, mean BMI = 26.40, 9.79 years old; 17 non-obese, BMI = 17.45, 9.76 years old) and thirty-one with high math skills (15 obese, BMI = 26.90, 9.60 years old; 16 non-obese, BMI = 17.13, 9.63 years old). No significant differences between weight groups were observed in task accuracy regardless of their mathematical skill level. For ERPs, electrophysiological differences were found only in the subtraction condition; participants with obesity showed an electrophysiologic pattern associated with a reduced ability to allocate attention resources regardless of their math skill level, these differences were characterized by longer P300 latency than their normal-weight peers. Moreover, the participants with obesity with high math skills displayed hypoactivity in left superior parietal lobule compared with their normal-weight peers. Additionally, obese preteens with low math skills displayed smaller arithmetic N400 amplitude than non-obese participants, reflecting difficulties in retrieving visual, semantic, and lexical information about numbers. We conclude that participants with obesity are less able than their normal-weight peers to deploy their attention regardless of their behavioral performance, which seems to have a greater effect on obese participants with low math skills because they also show problems in the retrieval of solutions from working memory, resulting in a delay in the development of mathematical skills.

## Introduction

Math skills entail adding, subtracting, multiplying, and dividing symbolic numbers efficiently, and are developed through childhood and adolescence (Siegler et al., 2011; Siegler and Braithwaite, 2017). These skills constitute an essential element of the K-12 school curriculum and have been positively associated with academic achievement (Gómez-Velázquez et al., 2015). Thus, failure to adequately develop such skills may have deleterious effects on an individual’s daily life.

Arithmetic fact processing involves basic and complex cognitive processes and develops over time (e.g., Geary et al., 2008; Geary, 2010; Meyer et al., 2010). Multiple studies have identified the basic cognitive processes which predict math skills, noting that cognitive inhibition, set-shifting (van der Sluis et al., 2004; Wei et al., 2018), working memory (Geary, 2010; Xenidou-Dervou et al., 2017; Wei et al., 2018), visuospatial skills, and executive functions (Kahl et al., 2021) explain children’s ability to solve arithmetic problems and incorporate arithmetic facts, suggesting that children require a higher order cognitive to reach certain math skills.

Recent studies have proposed a role for environmental factors, noting that sex (Hyde, 2016; Palomares-Ruiz and García-Perales, 2020), education (Palomares-Ruiz and García-Perales, 2020), or weight status also affect mathematical skill levels. Several studies have highlighted a negative relationship between body mass index (BMI) and math achievement scores (Crosnoe, 2004; Castelli et al., 2007; Cottrell et al., 2007; Manes, 2015). Further, longitudinal studies using standardized tests (e.g., Woodcock-McGrew-Werder mini-battery of achievement, Woodcock et al., 1994) reported a negative association between weight status and math scores (Datar et al., 2004; Gable et al., 2012; Kranjac, 2015; Li and O’Connelly, 2015; Martin et al., 2017). Kranjac (2015) reported that the math skills of children with obesity were independent of the level of self-efficacy (i.e., the ability to organize and execute behaviors needed to attain chosen goals, Bandura, 1997). Participants with obesity had lower scores by age than their normal-weight peers, indicating a direct negative effect of obesity on math skills.

Numerous studies have noted problems in executive functions such as inhibitory control (Mamrot and Hanć, 2019; Alatorre-Cruz et al., 2021), attention (Maayan et al., 2011; Blanco-Gómez et al., 2015; Ayoub-Meo et al., 2019), and working memory (WM, Maayan et al., 2011; Schwartz et al., 2013) in children with obesity. Neuroimaging studies have attributed these problems to decreased gray matter volume or density in multiple brain regions (Alosco et al., 2014; Borsini et al., 2015; Ou et al., 2015; Perlaki et al., 2018; Laurent et al., 2020; Ronan et al., 2020). Although the cognitive processes affected in children with obesity overlap those predicting math skills (van der Sluis et al., 2004; Geary, 2010; Xenidou-Dervou et al., 2017; Wei et al., 2018; Kahl et al., 2021), it is possible that there is an interaction between weight status and developmental age, (Siegler et al., 2011; Siegler and Braithwaite, 2017), which will affect the consolidation of cognitive processes associated with each arithmetic processing stage [i.e., encoding, math strategy, and verification (Campbell, 2005)].

Prior to consolidation of math skills, children must have an adequate association between mental representations and numerical magnitudes to calculate arithmetic facts [i.e., encoding stage (Dehaene and Cohen, 1995; Siegler et al., 2011; Siegler and Braithwaite, 2017; Malone et al., 2019)]. Moreover, there is a shift in the strategy used to solve problems in childhood, from a reliance on procedural strategies to a greater reliance on retrieval strategies (Cragg and Gilmore, 2014; Cragg et al., 2017). Most studies exploring arithmetical development have shown that strategy development is not an abrupt shift, but rather a change in the frequency of strategy choices used by children at different time points [overlapping waves theory (Siegler, 1996)], with some studies suggesting that the procedural strategy gradually evolves to one of retrieval (Geary, 2011; Peters and De Smedt, 2018). However, there is substantial evidence that strategy selection depends on both arithmetic development and the type of arithmetic problem (Zhou et al., 2006; Van Beek et al., 2014). For example, it is expected that children will solve addition or multiplication problems using a retrieval strategy, while subtraction will require procedural strategies (Zhou et al., 2006; Barrouillet et al., 2008; Van Beek et al., 2014). Development of an efficient strategy is reflected in the subject’s performance, particularly in response times (RTs) (Cragg et al., 2017; Proverbio and Caminati, 2019). For example, it has been suggested that young adults have a greater mastery of the resolution of arithmetical facts than children because young adults display shorter RTs, even when both age groups have the same response accuracy and trigger the same kind of math strategy (Cragg et al., 2017). We might suppose that when two children solving the same math problem differ in their RTs, they are using a different strategy or have different mastery levels using the same strategy.

Given that math strategy selection is not reflective of math skill level, event-related potential (ERP) studies have focused on specific components of mathematical problem solving such as the time at which the participant verifies a presented result, where that the individual’s math skill is reflected not only in the behavioral performance but also in the electrophysiological pattern. For example, Proverbio and Caminati (2019) reported that adults who were skilled math performers had shorter RTs, greater response accuracy and an electrophysiological pattern characterized by shorter latencies in a frontocentral positivity occurring between 300 and 500 ms (P300 component) than individuals with poorer math skills in arithmetic. Additionally, participants with poorer skills displayed larger P300 amplitude for incorrect than correct responses compared with the higher-performing participants. The P300 component is generally associated with attention allocation, and its amplitude is affected by increased task complexity (Polich, 2007). Proverbio and Caminati (2019) concluded that lower-performing participants had a poorer representation of arithmetic solutions and more difficulty retrieving that information because they were less able to reject incorrect solutions due to their posited solution strategy.

Based on the idea that adults have greater math skills than children, ERP studies comparing these age groups reveal that brain-electrical activity associated with arithmetic skills depends on age and the kind of arithmetical problem. Prieto-Corona et al. (2010) compared ERPs of children and adults during a multiplication task. Both age groups displayed a negativity between 250 and 450 ms in centro-parietal regions (arithmetic N400 component) and a late positive component (LPC) that appears between 500 and 675 ms. Group differences were observed in the arithmetic N400 component, with children displaying a larger amplitude and longer latency with a greater spatial distribution than adults. There is general agreement that arithmetic facts are stored in semantic networks (Kutas and Hillyard, 1980), with N400 amplitude modulations attributed to the relatedness or memory representations associated with the problem and its possible solutions (Niedeggen et al., 1999). Thus, Prieto-Corona et al. (2010) concluded that children displayed greater cognitive effort in retrieving the solution from long-term memory than adults. Following this line of thought, Zhou et al. (2011) compared adults and children on addition and multiplication tasks, finding different results; the arithmetic N400 was only observed for addition, while a left anterior negativity was reported for multiplication (N400, Kutas and Hillyard, 1980). The age groups differed in both ERP components, with children displaying smaller arithmetic N400 amplitude in the multiplication condition and greater arithmetic N400 amplitude in the addition condition than the adults. Based on their earlier work in adults Zhou et al. (2007), proposed that children rely more than adults on a quantitative manipulation supported on brain networks associated with manipulation of numerical magnitude while the smaller ERP amplitude during multiplication reflects a less stable verbal processing system for solving multiplication problems.

Problem complexity effects on age-related differences in performance include increased RTs and error rates as complexity increases, resulting in the split effect of strategy selection (Imbo and Vandierendonck, 2007; Van Beek et al., 2014). The split effect is defined as the use of retrieval strategies for simple problems and procedural strategies for more complex problems (Imbo and Vandierendonck, 2007). This effect is associated with an electrophysiological pattern in which adolescents have a greater frontocentral negativity between 250 and 500 ms (N280 component) for large than small additions and larger LPC in right sensors for small than large additions (Van Beek et al., 2014). In this study, the N280 component seems to serve a function similar to the arithmetic N400 component observed in adults. The greater N280 amplitude was interpreted to reflect greater engagement of attention and working memory resources for large than small addition, while the LPC amplitude was interpreted as a marker of the split effect.

Few studies have analyzed ERP components in children or preteens, and none have compared differences in mathematical processing in obese and normal weight, despite the recognized association between childhood obesity and poorer mathematical skills. It would be expected that obese and non-obese preteens have a consolidated mental representation and understanding of numerical magnitudes and an efficient strategy for solving arithmetic problems that includes appropriate use of the two types of math strategy. As childhood obesity has been associated with poor behavioral performance, a better understanding of the specific processing stages affected in this population is needed.

In this study, we assessed the math abilities of obese and non-obese preteens. We stratified participants by performance on a mathematical pre-test and then compared their performance, brain-electrical activity (ERPs), and ERP sources during the verification stage of addition and subtraction operations. We expected to find worse performance in participants with obesity than non-obese participants, with participants with obesity exhibiting lower response accuracy and longer RTs than their normal-weight peers. Three consistent ERP components were analyzed: the P150, P300, and arithmetic N400. We expected to find worse performance in participants with obesity than their normal weight peers, with electrophysiological responses consistent with greater cognitive effort, difficulties with attention allocation and with answer retrieval. Thus, we hypothesized that there would be differences between weight and mathematical skill groups in the P300, reflecting difficulty in allocating attentional resources, and N400, where longer latency and greater amplitude would reflect problems with answer retrieval. We did not expect to see differences between groups or conditions for the P150 component. We also expected that preteens with obesity with high or low math skills would show worse performance and an electrophysiological pattern associated with a greater cognitive effort than their normal-weight peers. Given that preteens between 9 and 10 years old should efficiently solve the simplest operations, such as addition, we expected differences between weight groups only in subtraction condition.

## Materials and Methods

### Participants

Sixty-two preteens were enrolled in this study {38 females and 24 males, age 9–10 years [mean (M) = 9.70 years, standard deviation, SD: 0.53 years]}. All participants completed at least the 3rd grade of schooling, were right-handed (Edinburgh Handedness Inventory, Oldfield, 1971), had normal or corrected to normal vision (assessed by Snellen Eye Chart), and normal hearing (20 dB assessed by a standard audiometer). The preteens parents also completed measures of their socioeconomic status (SES, measured by Four-Factor Index of Social Positions, Hollingshead, 1975). Thirty-three preteens were normal weight (between 5th and 90th BMI percentile; non-obese group), and twenty-nine were overweight (Above the 95th BMI percentile; obese group). Subjects were excluded if they had been diagnosed with a physical or mental health condition, if they were currently taking neuroactive medications, or if they had a head injury that resulted in loss of consciousness.

A certified psychological examiner provided cognitive assessments that included the Behavior Rating Inventory of Executive Functions (BRIEF, reliability coefficient (RC): 0.82 for parents, Gioia et al., 2000), Delis-Kaplan Executive Function System (D-KEFS, RC: 0.80, Delis et al., 2001), Children Memory Scale (CMS, average RC: 0.70, Cohen, 1997), Wechsler Abbreviated Scale of Intelligence (WASI-II, RC: 0.87–0.91, Wechsler, 2011), Wisconsin Card Sorting Test (WCST, RC: 0.90, Kongs et al., 2000), and Wide Range Achievement Test (WRAT-4, RC: 0.78–0.89, Wilkenson and Robertson, 2006). Participants were excluded if they scored lower than 80 on the WASI-II or less than 90 on the WRAT-4 test. Sleep was evaluated qualitatively by use of the Children’s Sleep Habits Questionnaire (CSHQ, RC: 0.47–0.93, Owens et al., 2000), and quantitatively the night before experimental testing using wrist actigraphy. Sleep the night prior to the experimental study of less than 6 h resulted in exclusion from the study. We used math computation score of WRAT-4 test to calculate the power analysis for our sample size [Effect size *d* = 0.70; α error = 0.05; Power (1−β error prob.) = 0.86]. The study protocol was approved by the University of Arkansas for Medical Sciences Institutional Review Board with written informed consent obtained from parents and assent from preteens.

### Experimental Design

Participants were provided a standard breakfast of a moderate glycemic index (around 50) to guarantee all preteens consumed the same nutritional components of the meal, and then they were assessed for mathematical skills using thirty complex addition and subtraction problems (CAS). The problems were characterized by three operands, with half requiring addition of the operands, and the other half of adding up two operands and subtracting the last (e.g., 8 + 10 + 3; 9 + 6 – 4). Following presentation of the problem three response options were provided and the subject was asked to select the correct response. Following this assessment, participants were prepared for the experimental task.

Preteens were seated comfortably on a padded chair with their back supported in a sound-isolating, shielded recording chamber. All participants were instrumented for EEG recording using the 128-channel Geodesic Sensor Net™ (Magstim EGI, Eugene, OR, United States). The stimuli were designed and administered using E-Prime software (Version 2). Stimuli were presented on a computer screen located approximately 60 cm from the participant. A practice block with 20 trials representative of the complete task, (10 additions and 10 subtractions), followed by two experimental blocks of 120 trials each for 240 trials was administered (120 additions and 120 subtractions). Stimuli were not reused between blocks, and practice stimuli were randomly chosen from experimental blocks. All arithmetic problems consisted of two operands which were randomized addition and subtraction questions. The participants had a short rest period after each experimental block, after which they were able to initiate the next block at their discretion.

**FIGURE 1:**
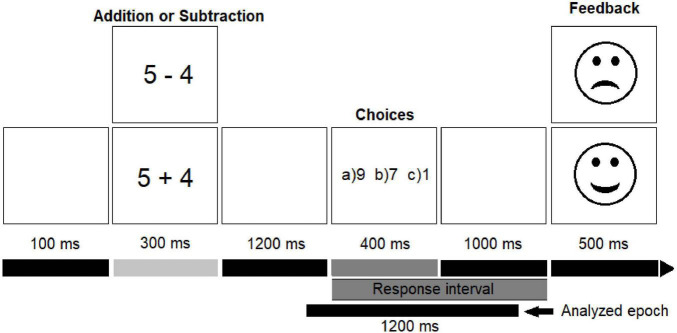
Paradigm of the delayed-verification math task.

All visual stimuli were black and presented centrally on a white background (Arial, 24 points). Each trial began with a blank screen displayed for 100 ms, presentation of arithmetic problems (e.g., 5 + 4) for 300 ms, followed by a blank screen for 1,200 ms. Three possible answers were then presented on the screen [e.g., (a) 11, (b) 9, (c) 7] for 400 ms, with the correct answer to the presented problem always included in the choices. Upon choice presentation, participants were given a response interval of 1,400 ms, and if no choice was selected in this time, “no response” was recorded (see Figure 1). Participants were asked to press the key corresponding to their chosen answer on a button box with the first three fingers of their right-hand. At the end of each trial, feedback was provided with a happy face denoting a correct response and an unhappy face associated with an incorrect response. The feedback appeared for 500 ms. Participants were instructed to respond as quickly and accurately as possible. The entire session, including EEG preparation, took approximately 1.5 h.

### Data Acquisition/Pre-processing

The electroencephalogram (EEG) was acquired with a Geodesic Net Amps 300 system running NetStation 4.5.2 software using the 128-channel Geodesic HydroCel Sensor Net™ (Magstim EGI, Eugene, OR, United States). The EEG signals were amplified, low pass filtered at 100 Hz and digitized with a sampling rate of 500 Hz. Electrode impedances were kept below 50 kΩ. Data were analyzed off-line using the open-source EEGLAB toolbox (MATLAB version R2020a, EEGlab v. 2019.1) (Delorme and Makeig, 2004). Data were re-referenced offline to the common mean and band-pass filtered at 0.1–57 Hz. Eye movements, blinks, and heart rhythm were excluded using independent component analysis. We excluded those components with wave morphology and topographical location commonly associated with EEG artifacts. Bad channels were interpolated from nearby good channels using spherical splines. EEG included had less than ten interpolated channels.

#### Event-Related Potential Data

Event-related potentials were time-locked to presentation of the answer choices. Data were segmented with a 200 ms pre-stimulus baseline, and 1,000 ms post-stimulus duration. The resultant segments were subjected to an automatic-artifact detection algorithm; segments where one or more channels had zero variance or a channel amplitude exceeding 100 μV were excluded from analysis. Following this, segments were visually inspected by an experimenter who was blind to experimental group, and those with less than forty percent of artifact-free segments by experimental condition were rejected (On average 54 segments of addition condition and 50 of subtraction condition were accepted). The percentage of accepted segments was not significantly different between groups [addition: *t*(60) = −0.30, *p* = 0.76; subtraction: *t*(60) = −0.90, *p* = 0.93]. Segments were baseline corrected based using the pre-stimulus time window and averaged in each experimental condition (i.e., addition and subtraction) for each participant. Averaged waveforms included only correctly answered trials. A collapsed localizer approach was used to identify the time windows of ERPs components, and this approach consisted of averaging all experimental conditions (Luck and Gaspelin, 2017).

### Data Analyses Methods

#### Clustering Analysis to Determinate Math Skill Level

We determined the participant-specific math skill level for each weight group using a K-means clustering. The variables included in this analysis were the CAS score and math task response: accuracy and RT for both experimental conditions. Clustering resulted in four subgroups: obese participants with low or high math skills, and non-obese participants with low and high math skills. After the clustering analysis, we compared CAS score between groups using three-way ANOVAs. Weight group and math skill level were included as a between-subjects factor, with CAS score included as a within-subjects factor. The effect size for each significant comparison was calculated and reported as Cohen’s *d*.

#### Demographic Data

Three-way ANOVAs were used to compare demographic data between groups. In all comparisons, weight group and math skill level were included as a between-subjects factor, and age, SES score, BMI, or BMI percentile were included as within-subjects factors. Cohen’s *d* was reported for each significant comparison. Group composition for sex was evaluated using a Chi-squared test.

#### Behavioral Data in Math Task

Three-way ANOVAs were separately computed for each math task performance metrics (i.e., response accuracy and RT), these comparisons were performed separately for each experimental condition (addition and subtraction). Before analyses, the response accuracy was transformed using the function {ARCSINE [Square Root (percentage/100)]} to ensure a normal distribution of the data (Zar, 1998). In all comparisons, weight group and math skill level were included as a between-subjects factor, with response accuracy or RTs of each experimental condition (i.e., addition or subtraction) included as a within-subjects factor. Cohen’s *d* was reported for each significant comparison.

#### Psychometric Tests

Psychometric test scores (BRIEF, CMS, WASI-II, WCST, and WRAT-4) were compared between weight groups using three-way ANOVAs. Weight group and math skill level were evaluated as a between-subjects factor. Table 1 shows the indices and subscales comprising the within-subject factors and the cognitive functions they assess. Cohen’s *d* was reported for each significant comparison.

**TABLE 1:** Psychometric test measures included as between-subject factor.

Psychometric test	Description	Between-subject factor
BRIEF	Assesses executive function in school and home environments and is composed of two broad indices	Behavioral regulation index
		Metacognition index
CMS	Evaluates memory and learning	Visual immediate
		Visual delayed
		Verbal delayed
		Verbal immediate
		General memory
		Learning
		Delayed recognition
WASI-II	Assesses intelligence	Vocabulary
		Block design
		Similarities
		Matrix reasoning
WCST	This test is used to measure executive functions	Perseverative response
		Perseverative error
		Non-perseverative errors
WRAT-4	Assesses academic skills	Reading
		Spelling
		Math computation
D-KEFS^1^	This is neuropsychological battery to assess executive functions	Trail making (TM)
		-Visual scanning
		-Number sequencing
		-Letter sequencing
		-Number letter switching
		Verbal fluency (VF)
		-Letter fluency
		-Category fluency
		-Category switching
		Color word (CW)
		-Color naming
		-Word Reading
		-Inhibition
		-Inhibition switching

*BRIEF, Behavior Rating Inventory of Executive Functions; CMS, Children Memory Scale; WASI-II, Wechsler Abbreviated Scale of Intelligence; WCST, Wisconsin Card Sorting Test; WRAT-4, Wide Range Achievement Test-4; D-KEFS, Delis-Kaplan Executive Function System.*

*^1^We performed three-way ANOVAs for each subtest (i.e., TM, VF, and CW).*

#### Electrophysiological Data

##### Amplitude and Latency Analyses

Grand averaged ERPs showed positivity between 100 and 200 ms (P150 component), followed by a positivity between 200 and 400 ms (P300 component, Polich, 2007) on frontal regions and a negative wave between 300 and 500 ms on central parietal regions (arithmetic N400) (Niedeggen et al., 1999; Prieto-Corona et al., 2010; Zhou et al., 2011). We analyzed only the ERPs components with a more robust signal observed in both addition and subtraction regardless of group. The mean amplitude for each component (P150, P300, and arithmetic N400) and its latency assessed as time-to-peak amplitude were analyzed.

**FIGURE 2:**
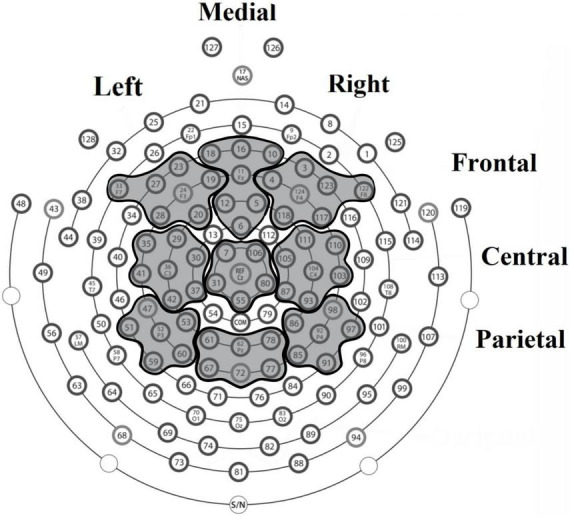
Electrode arrangement used for amplitude and latency analyses of ERP components.

We explored specific region of interest (ROIs) for each ERP component based on the topographical locations associated with these ERP components in previous literature (e.g., Niedeggen et al., 1999; Polich, 2007; Prieto-Corona et al., 2010; Zhou et al., 2011). For P150 and P300 components three ROIs were compared between weight groups: right frontal (sensors 3, 4, 117, 118, 122, 123, and 124), left frontal (sensors 19, 20, 23, 24, 27, 28, and 33), and fronto-central (sensors 5, 6, 10, 11, 12, 16, and 18); for the arithmetic N400 component we analyzed six anatomical regions: right central (sensors 87, 93, 103, 104, 105, 110, and 111), left central (sensors 29, 30, 35, 36, 37, 41, and 42), central (6, 7, 31, 55, 80, 106, and Cz), right parietal (sensors 85, 86, 91, 92, 97, and 98), left parietal (sensors 47, 51, 52, 53, 59, and 60), centro-parietal (61, 62, 67, 72, 77, and 78) (see Figure 2).

Three-way ANOVAs were separately computed for each experimental condition (i.e., addition or subtraction), for each ERP component and each ROI (sensors), with weight group and math skill level as the between-subject factors, and amplitude or latency of ERPs components in each ROI as the within-subject factor. Tukey’s HSD method was used for *post hoc* pairwise comparisons. Data were analyzed using SPSS Statistics 20. Greenhouse–Geisser corrections were made for violations of sphericity when the numerator was greater than 1. *P*-values resulting from a set of comparisons were corrected by the false discovery rate method (FDR), only significant *p*-values after FDR were reported.

##### Source Analyses

Electrical source analysis was computed using sLORETA version 20200414 (Pascual-Marquis, 2002) for P300 and arithmetic N400 components. The cortex was modeled as a collection of volume elements (6,239 voxels, size 5 × 5 × 5 mm) in digitized Montreal Neurological Institute (MNI) coordinates. Electrode coordinates were generated from 128 channels (Magstim EGI, Eugene, OR, United States). A transformation matrix was created using electrode coordinates and sLORETA values for each participant.

Two statistical comparisons were made: first, weight group was evaluated by math skill level and second, we compared each weight by math skill group separately for our two experimental conditions (subtraction and addition). Statistical analyses were performed using a t-statistic for independent groups with 5,000 random permutations for each ERP component (i.e., P150, P300, and arithmetic N400) and resultant values were visualized on a three-dimensional brain model and evaluated for significances (*p*-value < 0.05 considered significant). Additionally, we report the maximum differences between groups at the respective MNI coordinates and Brodmann areas (BA).

##### Correlations Analyses

Correlation analysis was used to evaluate the relationship between neuropsychological scores (BRIEF, D-KEFS, CMS, WASI-II, WCST, and WRAT-4 tests), math task performance metrics (accuracy and RTs for addition or subtraction conditions), and significant differences in electrophysiological data (mean amplitude or latency of P300 and arithmetic N400 components in each ROI).

## Results

### Clustering Analyses Results of Preteens’s Math Skill Level

**FIGURE 3:**
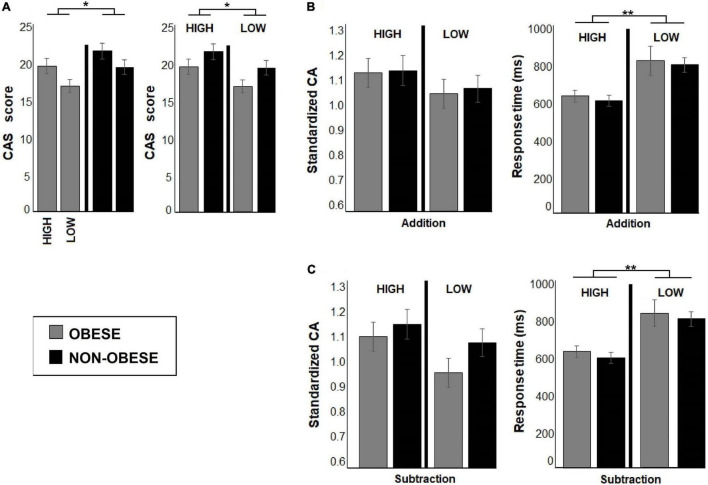
The bar graphs show behavioral performance during addition and subtraction conditions for each subgroup after clustering analyses. In **(A)** bar graphs show the differences between weight groups and math skill levels in CAS scores, while **(B,C)** illustrate accuracy and response times (RTs) in math task for addition and subtraction conditions for all subgroups. Both bar graphs show differences between preteens with high and low math skill level in RTs. The preteens with low math skills displayed longer RTs than the high performers in both experimental conditions. Significant *p*-values were represented as follows: *p < 0.05, **p < 0.001.

Cluster analysis resulted in identification of four weight by math skill groups: (1) 14 participants with obesity and low math skills, (2) 15 preteens with obesity and high math skills, (3) 17 non-obese preteens with low math skills, and (4) 16 non-obese preteens with high math skills. Significant main effect of weight group [*F*(1,58) = 4.37, *p* = 0.04, Cohen’s *d* = −0.50] and math skill level [*F*(1,58) = 4.96, *p* = 0.03, Cohen’s *d* = −0.53] were observed. The non-obese preteens had higher CAS scores than participants with obesity (Obese, *M* = 18.91; non-obese, *M* = 21.30), and higher CAS scores were found for preteens with high math skill level than those with lower performance (Low, *M* = 18.83; high, 21.38). No significant weight group by math skill level interaction in CAS scores was observed (see Figure 3A).

### Demographic Results

No differences between weight groups or math skill level in age or SES score were found. As expected, significant main effect of weight group in BMI [*F*(1,58) = 144.88, *p* < 0.001, Cohen’s *d* = 3.00] and BMI percentile [*F*(1,58) = 94.58, *p* < 0.001, Cohen’s *d* = 2.60] were seen. The preteens with obesity showed higher scores in both measures than their normal-weight peers (see Table 2). No weight group by math skill level interaction was statistically significant in any comparison. The weight groups or participants with low or high math skill level did not differ in gender [Weight group, χ^2^ (1) = 0.91, *p* = 0.56; math skill level, χ^2^ (1) = 0.29, *p* = 0.21].

**TABLE 2:** Demographic data of preteens.

		Weight status	
		Obese	Non-obese
		
	Math skill level	Mean (SD)	
Age	Low	9.79 (0.58)	9.76 (0.44)
	High	9.60 (0.51)	9.63 (0.62)
SES score	Low	38.42 (9.90)	45.88 (10.70)
	High	47.10 (7.97)	46.56 (9.74)
BMI	Low	26.40 (5.10)	17.45 (1.55)
	High	26.90 (2.97)	17.13 (1.65)
BMI percentile	Low	96.73 (2.55)	53.94 (23.69)
	High	97.63 (1.89)	51.16 (25.46)

		**Male/Female**	

Sex	Low	5/9	5/12
	High	6/9	8/8

*SD, standard deviation; BMI, body mass index; SES, socioeconomic status; Low, low math skills; High, high math skills.*

### Behavioral Results of Math Task

#### Addition

As shown in Figure 3B, no differences in response accuracy between weight groups or math skill level were observed. However, a main effect of math skill level was found in RT [*F*(1,58) = 98.02, *p* < 0.000, Cohen’s *d* = 2.50]. Preteens with low math skills had longer RT than those with high math skills (see Table 3). No significant weight group by math skill level interaction was found.

**TABLE 3:** Behavioral results of math task performance.

		Addition		Subtraction	
Weight status	Math skill level	A (SD)	RTs (SD)	A (SD)	RTs (SD)
Obese	Low	1.06 (0.21)	850.64 (77.37)	0.97 (0.28)	864.00 (80.82)
Non-obese		1.08 (0.20)	826.41 (86.11)	1.86 (0.20)	819.94 (89.86)
Obese	High	1.13 (0.22)	651.87 (72.22)	1.11 (0.23)	648.27 (70.96)
Non-obese		1.15 (0.23)	626.12 (79.06)	1.15 (0.26)	615.75 (72.84)

*A, accuracy; RT, response times; SD, standard deviation; Low, low math skills; High, high math skills.*

#### Subtraction

There were no differences between weight groups or math skill levels in response accuracy (see Figure 3C), but the RT depended on math skill level [*F*(1,58) = 108.24, *p* < 0.000, Cohen’s *d* = 2.60]. The participants with low math skill level displayed longer RT than preteens with high math skill level regardless of weight status (see Table 3). In these comparisons, no significant weight group by math skill level interaction was found.

### Psychometric Tests

Differences in psychometric tests were observed between weight groups regardless of math skill level in TM subtest of the D-KEFS [*F*(1,57) = 12.89, *p* = 0.001, Cohen’s *d* = 0.69] and WRAT-4 test [*F*(1,56) = 9.84, *p* = 0.003, Cohen’s *d* = 0.68], with participants with obesity exhibiting lower scores than their normal-weight peers (TM subtest, obese, *M* = 8.89; non-obese, *M* = 10.81; WRAT-4 scores, obese, *M* = 105.98; non-obese, *M* = 112.77). TM assesses executive functions, particularly cognitive flexibility in a visual-motor sequencing task. A main effect of math skill level was observed [*F*(1,56) = 11.03, *p* = 0.003, Cohen’s *d* = 0.71] in the WRAT-4 test, where participants with high math skills had higher WRAT-4 scores than those with low math skills (Math skill level: low, *M* = 105.78; high, *M* = 112.97). No weight group by math skill level interaction was statistically significant (see Table 4).

**TABLE 4:** Psychometric assessment of preteens.

			Main effect		Cohen’s *d*
	Obese	Non-obese	Weight group	Math skills	G/M
**Psychometric tests**	**Mean (SD)**		***p*-value**		
BRIEF (Indices)			0.90	0.31	0.03/0.15
-BRI	46.71 (11.74)	46.56 (9.46)			
-MI	48.88 (11.20)	48.34 (10.03)			
CMS (Indices)			0.20	0.67	0.25/0.08
-Visual immediate	97.99 (17.53)	100.58 (8.37)			
-Visual delayed	98.54 (13.23)	101.62 (12.63)			
-Verbal immediate	102.54 (18.35)	107.87 (15.53)			
-Verbal delayed	102.83 (15.13)	106.28 (12.44)			
-General memory	101.32 (17.61)	106.86 (12.05)			
-Learning	97.91 (13.53)	102.14 (12.20)			
-Delayed recognition	102.93 (11.66)	102.94 (14.08)			
D-KEFS					
-TM**			0.001	0.59	0.68/0.10
Visual scanning	8.03 (2.93)	10.75 (2.70)			
Number sequencing	9.61 (2.52)	11.27 (2.52)			
Letter sequencing	9.16 (3.04)	10.49 (2.62)			
Number letter switching	8.75 (2.93)	10.76 (2.50)			
-VF			0.06	0.52	0.38/0.13
Letter fluency	9.50 (2.15)	10.22 (2.56)			
Category fluency	10.45 (2.00)	11.41 (2.75)			
Category switching	10.24 (2.46)	11.33 (2.87)			
-CW					
Color naming	10.31 (2.57)	10.65 (2.60)	0.31	0.10	0.22/0.36
Word reading	10.37 (1.97)	11.04 (2.02)			
Inhibition	9.80 (2.61)	11.04 (2.69)			
Inhibition switching	10.48 (2.31)	10.38 (2.84)			
WASI-II (subtests)			0.26	0.40	0.17/0.16
-Vocabulary	55.67 (8.29)	54.02 (9.61)			
-Block design	48.13 (10.26)	51.10 (9.29)			
-Similarities	52.49 (8.82)	53.94 (9.00)			
-Matrix reasoning	50.41 (7.75)	55.33 (6.91)			
WCST			0.14	0.79	0.32/0.28
-Perseverative responses	109.05 (23.75)	118.40 (23.32)			
-Perseverative errors	110.40 (21.80)	110.41 (18.73)			
-Non-perseverative errors	108.48 (23.53)	120.35 (22.67)			
WRAT-4 (subtests)*			0.002	0.003	0.68/0.71
-Reading	104.01 (6.56)	108.62 (7.21)			
-Spelling	108.20 (11.26)	116.37 (12.02)			
-Math computation	105.74 (10.80)	113.32 (11.97)			

*G, main effect of weight group; M, main effect of math skills; SD, standard deviation; BRIEF, Behavior Rating Inventory of Executive Functions; BRI, Behavioral regulation index; MI, Metacognition index; CMS, Children Memory Scale; WASI-II, Wechsler Abbreviated Scale of Intelligence; WCST, Wisconsin Card Sorting Test; WRAT-4, Wide Range Achievement Test-4; D-KEFS, Delis-Kaplan Executive Function System; TM, trail making; VF, verbal fluency, CW, color word.*

Significant p-values were represented as follows: *p < 0.05, **p < 0.001.

### Analyses of Event-Related Potentials

#### Amplitude and Latency

##### Addition

There were no significant differences between weight or math skill level groups in the amplitude or latency of ERPs components. No weight group by math skill level interaction was statistically significant.

##### Subtraction

**FIGURE 4:**
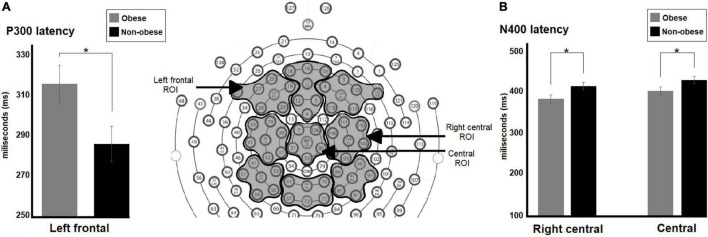
Differences between preteens with obesity and no-obese preteens regardless math skill level in latency of P300 and arithmetic N400 components. In **(A)** the bar graph shows differences between weight groups in P300 latency in left frontal ROI, the preteens with obesity displayed longer P300 latency than their normal weight peers. In **(B)** the bar graph illustrates differences between weight groups in N400 latency in right central and central ROIs. The participants with obesity showed shorter N400 latency than the other group in both ROIs. Significant *p*-values were represented as follows: *p < 0.05.

Comparison between preteens revealed no significant differences in P150 amplitude or latency between weight groups or math skill level for any ROI analyzed. However, weight groups differed in latency of P300 and arithmetic N400 components regardless of math skill level. A significant main effect of weight groups in P300 latency in the left frontal ROI was seen [*F*(1,58) = 5.32, *p* = 0.02, Cohen’s *d* = 0.44]. The participants with obesity showed longer P300 latency than their normal-weight peers (Obese, *M* = 315.14 ms; non-obese, *M* = 285.86 ms) (see Figure 4A).

There were significant main effects of weight group in arithmetic N400 latency in right central [*F*(1,58) 5.42, *p* = 0.02, Cohen’s *d* = −0.46] and central [*F*(1,58) = 4.10, *p* = 0.05, Cohen’s *d* = −0.38] ROIs. The preteens with obesity showed shorter arithmetic N400 latency than their normal-weight peers in both ROIs (Right central, obese, *M* = 384.96 ms; non-obese, *M* = 416.67 ms; central, obese, *M* = 431.0 ms; non-obese, *M* = 404.81 ms) (see Figure 4B).

**FIGURE 5:**
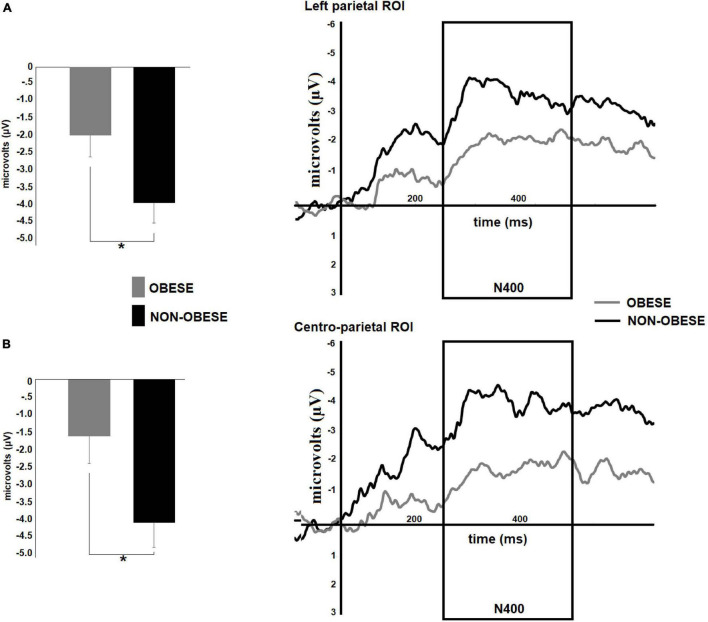
Differences between obese and no-obese preteens with low math skills in arithmetic N400 amplitude. Right panels show the mean amplitude maps of the arithmetic N400 component in left parietal and centro-parietal regions for both weight groups during subtraction condition. The bar graphs **(A,B)** illustrate the differences between groups in arithmetic N400 amplitude in each region of interest: **(A)** left parietal, and **(B)** centro-parietal ROIs. A greater N400 amplitude was observed for non-obese participants. Significant *p*-values were represented as follows: *p < 0.05.

The statistical analyses also showed significant weight group by math skill interactions in arithmetic N400 amplitude in left parietal [*F*(1,58) = 4.65, *p* = 0.03] and parieto-central [*F*(1,58) = 4.71, *p* = 0.03] ROIs (see Figure 5). For both comparisons, *post hoc* tests revealed significant differences between weight groups only for participants with low math skill level [Left parietal: low, MD = 1.98, *p* = 0.03; high, MD = 0.75 *p* = 0.40; centro-parietal: low, mean difference (MD) = 2.53, *p* = 0.04; high, MD = 1.20, *p* = 0.33]. The non-obese participants showed greater arithmetic N400 amplitude than preteens with obesity in both ROIs (Left parietal, obese, *M* = −1.97 μV; non-obese, *M* = −3.95 μV; centro-parietal, obese, *M* = −1.66 μV; non-obese, *M* = −4.19 μV).

#### Source Analyses of Event-Related Potentials

**FIGURE 6:**
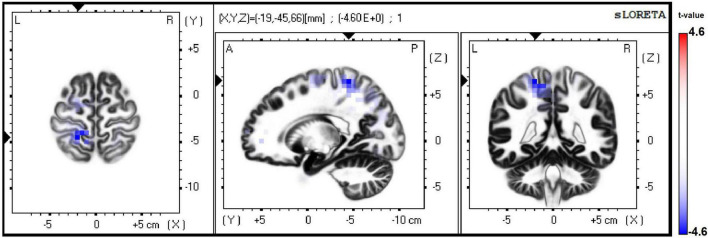
Differences in source analyses of ERP in subtraction condition between weight groups with high math skills. In blue hypoactivation of the left superior parietal lobule (BA 5).

Although source analyses showed no differences between obese and non-obese participants with low math skills in any ERP component or experimental condition, differences between weight groups with high math skill level were seen in ERP source analysis in the subtraction condition. Source localization of the P300 component indicated a hypoactivation in left superior parietal lobule (BA 5) in obese compared to non-obese participants (tmax = −4.57, *p* = 0.05) (see Figure 6).

### Correlation Analyses

**FIGURE 7:**
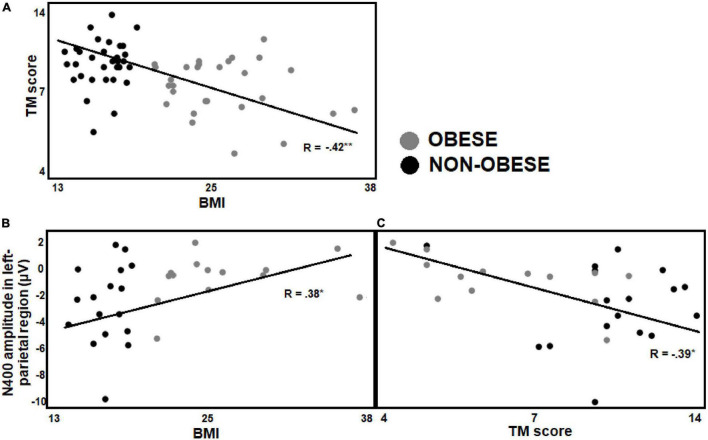
Pearson correlations between BMI, behavioral and electrophysiological data. **(A)** Illustrates the Pearson correlations between BMI and TM scores in the preteens regardless of math skills level, the BMI negatively correlated with TM scores. **(B,C)** Show Pearson correlations in the preteens with low math skills. **(B)** Illustrates a positive correlation between BMI and N400 amplitude in left parietal ROI, and **(C)** a negative correlation between TM score and N400 amplitude in this same ROI. Significant *p*-values were represented as follows: *p < 0.05, **p < 0.001.

As shown in Figure 7A, a negative correlation was found between BMI and TM scores (*R* = −0.42, *p* = 0.000) in preteens regardless of math skills level, preteens with greater BMI showed lower TM scores. In preteens with low math skills level, a positive correlation was observed between BMI and arithmetic N400 amplitude in left parietal (*R* = 0.38, *p* = 0.03) ROI, suggesting a smaller arithmetic N400 amplitude as BMI increases (see Figure 7B). In addition, the TM scores negatively correlated with the arithmetic N400 amplitude in left parietal ROI (*R* = −0.39, *p* = 0.03), that is, those participants with lower TM scores showed smaller N400 amplitude in both regions (see Figure 7C).

## Discussion

The goal of this study was to identify behavioral and electrophysiological differences between obese and non-obese participants with low or high math skill levels. Given that obesity has been associated with poorer mathematical skills, we hypothesized that obese preteens would exhibit worse behavioral performance and greater amplitude and longer latency of P300 and arithmetic N400 components on simple math problems associated with more cognitive effort in attention allocation and answer retrieval in both math skills level. Behaviorally, we observed no differences in accuracy or RT during the experimental math task between obese and non-obese participants, which is contrary to our hypothesis. One explanation for this result is that our math task did not require significant cognitive effort, given that all participants performed at a high level. Alternatively, in accord with the diffusion model, longer RTs may be used as a strategy to improve accuracy (Ratcliff and Smith, 2004; Starns and Ratcliff, 2010). Our experimental design provided all participants with sufficient time to calculate and verify their response, which may have positively affected their behavioral performance and reduced our ability to identify differences between groups.

While differences were not seen in task performance, there were significant differences between preteens with obesity and their normal-weight peers on psychometric tests. Regardless of math skill level, differences in psychometric tests favored the non-obese group. This finding is in accord with previous studies describing poorer executive functions (Maayan et al., 2011; Blanco-Gómez et al., 2015; Ayoub-Meo et al., 2019; Alatorre-Cruz et al., 2021) and lower math skills in children with obesity (Crosnoe, 2004; Castelli et al., 2007; Cottrell et al., 2007; Manes, 2015). Our study adds to this literature by indicating that these differences persist even in obese preteens with higher math skills.

In electrophysiological results, we hypothesized there would be differences between weight groups in the most reported ERPs; P300 and the arithmetic N400 components. In keeping with these expectations, we consistently observed a positivity between 200 and 400 ms on fronto-central locations, which we identify as the P300 (Polich, 2007). In our task, we suggest that the P300 component is associated with attentional processing as described in adults (Proverbio and Caminati, 2019). We further propose that this component could be associated with attention allocation (Dimoska et al., 2006; Ramautar et al., 2006; Polich, 2007) as our task requires preteens to evaluate three different responses and to select the correct response while inhibiting their response to incorrect answers. In addition, we also observed a negative wave between 300 and 500 ms on central and parietal ROIs in both experimental conditions (i.e., addition and subtraction), which we identify as the arithmetic N400 component.

As predicted, we found differences between weight groups in latency, amplitude, and source analyses of ERP components in the subtraction condition, with some of these differences dependent on the participant’s level of math skill. We expect that preteens have mastered simple addition, but that they would still be learning how to solve subtraction problems efficiently. Our findings are consistent with the expectation that they are gaining new skills in subtraction while evincing mastery over simple addition problems. If mastery of simple addition problems is considered a developmental milestone, then obese preteens seem to have passed this milestone. However, this group may face new mathematical challenges differently than their healthy-weight peers, as suggested by longitudinal studies in which participants with obesity seem to differ from their normal weight peers in arithmetical development throughout childhood and adolescence (Datar et al., 2004; Gable et al., 2012; Kranjac, 2015; Li and O’Connelly, 2015; Martin et al., 2017). Our finding that performance on subtraction problems in obese preteens was dependent on math skill level is novel and provides new information on differences in math performance between participants with obesity and normal weight preteens.

In preteens with obesity, we found that weight status negatively correlated with attentional control, with obese groups exhibiting longer P300 latency than their normal-weight peers in the subtraction condition regardless of math skill level. Longer P300 latency has been associated with a lower capacity to allocate attention (Dimoska et al., 2006; Ramautar et al., 2006; Polich, 2007). Therefore, these findings support the idea that executive functions are affected in the obese population (Maayan et al., 2011; Blanco-Gómez et al., 2015; Ayoub-Meo et al., 2019; Alatorre-Cruz et al., 2021). Moreover, this result match with Proverbio and Caminati’s (2019)’ finding. They found that participants with poorer math skills displayed longer P300 latency during incorrect and correct responses, which was not observed in participants with higher math skills, such modulation was interpreted as a poorer representation of arithmetic solutions and more difficulty retrieving that information. Thus, we can add to the interpretation that a longer P300 latency in obese preteens is associated with poorer representation of arithmetic solutions and more difficulty retrieving that information. It may also be reflecting a poorer attention control in the identification of correct solutions.

Based on the working memory system model (Baddeley, 2003), attention control and working memory are successive steps in complex processing tasks such as learning. Therefore, difficulties in one processing step will affect the next step. We thus suppose that problems in attention control will affect the retrieval of solutions from working memory and be reflected in greater amplitude and longer latency of arithmetic N400 component. Contrary to our hypothesis, we found shorter arithmetic N400 latency in right central and central ROIs for participants with obesity regardless of math skill level. Previous studies reported that in arithmetical processing, left areas are recruited when the problem requires subject calculates the result, while right and left areas are activated when a task’s mental demand is higher (Arsalidou et al., 2018). Therefore, we suggest that the longer arithmetic N400 latency in right-brain regions observed in participants with obesity might reflect that subtraction condition demands high neural resources to left hemisphere, thus they must recruit additional right-brain areas to retrieve the solutions from working memory.

Regarding the differences between weight groups that depended on math skill level, our findings were also unexpected. In participants with low math skill level, a smaller arithmetic N400 amplitude for obese than non-obese participants in left parietal and centro-parietal ROIs. An explanation for this result is that our findings did not match previous studies because we used a different experimental paradigm. These studies reported: (1) greater arithmetic N400 amplitude associated with a greater cognitive effort recovering an unrelated response (Niedeggen et al., 1999; Prieto-Corona et al., 2010), and (2) smaller arithmetic N400 amplitude associated with increasing age (Prieto-Corona et al., 2010; Zhou et al., 2011). Moreover, comparisons between children and adults might reflect the development of brain areas specializing in math processing. Adults are expected to require fewer neural resources than observed in children, resulting in a smaller arithmetic N400 amplitude (For review Peters and De Smedt, 2018).

In our study, we compared same-age preteens with the assumption that they were at a similar stage of cognitive development. Hence, we suggest that the differences we report between weight groups are related to the ability to efficiently activate neural networks associated with math skill processing. In children with poor math skills, neuroimaging studies have reported decreased network activation (Berteletti et al., 2014), and problems in modulation of brain responses as the complexity of arithmetic problems is increased (De Smedt et al., 2011; Ashkenazi et al., 2012). The electrophysiological pattern we report in obese preteens might reflect reduced neural-network activation during the processing of the retrieval of solutions from working memory. In addition, this possible explanation might also be supported on the topographical location of the arithmetic N400 component where the weight groups differed. Given that: (1) greater arithmetic N400 amplitude was observed in the left parietal region for non-obese participants, (2) greater arithmetic N400 amplitude was correlated with higher executive functions scores (i.e., TM scores), and (3) left brain areas should be recruited when the arithmetic problem is easy (Arsalidou et al., 2018). We suggest that non-obese participants might be spending more brain resources in the left hemisphere because they did not recruit additional areas as obese participants did to solve a complex problem. Therefore, differences between weight groups observed arithmetic N400 components may reflect problems in the retrieval of solutions from working memory for the participants with obesity.

In participant with high math skill level, we did not find differences between weight groups in the behavioral performance of our math task. Our weight groups only differed in source-level activation of the P300 component, with the obese group showing a hypoactivation of the left SPL. The posterior parietal cortex supports visuospatial or attentional processing (Vandenberghe and Gillebert, 2009), and plays an important role in the manipulation of visual-spatial or auditory-verbal information in working memory (Koenigs et al., 2009). For arithmetic processing, SPL is associated with three circuits for number processing. In particular, the SPL supports attentional orientation of the mental number line (Dehane et al., 2003; Arsalidou et al., 2018). The function of this brain area in arithmetic processing is still discussed, but it has been proposed that parietal, temporal, and occipital areas play an important role in the representation of figurative/object schemes during the calculation of arithmetic facts (Pascual-Leone and Johnson, 2005), with right parietal areas activated when a task’s mental demand is too high for the left hemisphere to trigger automatic schemas, while left parietal areas recruited when the problem require that the subject calculates the result, involving the child’s mental attentional capacity, thus a harder-relational figurative knowledge (Arsalidou et al., 2018).

In our study, we presume that subtraction operations require greater attentional orientation of the mental number line and thus harder-relational figurative knowledge, therefore, the preteens should recruit left parietal areas. The hypoactivation on left SPL observed in our participants with obesity may reflect a difficulty in brain response modulation with the increased arithmetical complexity of subtraction problems as is observed in children with poorer math skills or dyscalculia (De Smedt et al., 2011; Ashkenazi et al., 2012), which may be associated with an inefficient allocation of attentional orientation and weaker-relational schemes to solve the subtraction operation. We suggest that these results support the need for additional studies specifically focused on attentional resource allocation in obese adolescents during problem solving tasks.

## Conclusion

Our behavioral and electrophysiological results might indicate that lower mathematical skills previously reported in obese preteens are not directly associated with weight status. Rather, weight status seems to interact with the cognitive processes needed for math processing.

Interestingly, regardless of math skill level, differences between preteens with obesity and their normal-weight peers were related to the allocation of attentional resources and working memory processes. Thus, obese preteens seem to have worse executive control to solve math problems than their normal-weight peers, supporting the dependency relationship between weight status and these cognitive processes.

Our results support previously reported results in suggesting that participants with obesity face a disadvantage with respect to their normal-weight peers that is reflected in a delay in the development of math skills. This disadvantage may be due to poorer attention control and the retrieval of solutions from working memory that could be compensated by other cognitive processes, additional training, or recruitment of additional brain areas.

### Limitations

There are inherent limitations in the present study. No previous electrophysiological studies have assessed mathematical abilities between preteens with obesity and their normal-weight peers; therefore, our explanations are mainly based on findings observed in children or adults without a previous weight status classification. In this study, the low demand for speeded math processing may influence performance and prevent us from observing effects of obesity on response accuracy.

In addition, our sample size selection was not based on a prior study because no previous study has compared non-obese preteens and participants with obesity with high and low math skills. This fact may be a limitation, we suppose that differences between obese and non-obese preteens with high or low math skills could be stronger by recruiting a larger sample.

## Data Availability Statement

The original contributions presented in the study are publicly available. This raw data can be found here: https://openneuro.org/datasets/ds004019.

## Ethics Statement

The studies involving human participants were reviewed and approved by the University of Arkansas for Medical Sciences Institutional Review Board. Written informed consent to participate in this study was provided by the participants’ legal guardian/next of kin.

## Author Contributions

GA-C, SS, and LL-P contributed to the conception and design of the study. HD and DH organized the database and applied the experimental task. GA-C and DW performed the statistical analysis. GA-C and LL-P wrote the first draft of the manuscript. All authors contributed to manuscript revision, read, and approved the submitted version.

## Conflict of Interest

The authors declare that the research was conducted in the absence of any commercial or financial relationships that could be construed as a potential conflict of interest.

## Publisher’s Note

All claims expressed in this article are solely those of the authors and do not necessarily represent those of their affiliated organizations, or those of the publisher, the editors and the reviewers. Any product that may be evaluated in this article, or claim that may be made by its manufacturer, is not guaranteed or endorsed by the publisher.
